# Expression of Immuno-Oncologic Biomarkers Is Enriched in Colorectal Cancers and Other Solid Tumors Harboring the A59T Variant of *KRAS*

**DOI:** 10.3390/cells10061275

**Published:** 2021-05-21

**Authors:** Emil Lou, Joanne Xiu, Yasmine Baca, Andrew C. Nelson, Benjamin A. Weinberg, Muhammad Shaalan Beg, Mohamed E. Salem, Heinz-Josef Lenz, Philip Philip, Wafik S. El-Deiry, W. Michael Korn

**Affiliations:** 1 Division of Hematology, Oncology, and Transplantation, Department of Medicine, University of Minnesota, Minneapolis, MN 55455, USA; 2 Caris Life Sciences, Phoenix, AZ 85040, USA; jxiu@carisls.com (J.X.); ybaca@carisls.com (Y.B.); wmkorn@carisls.com (W.M.K.); 3 Laboratory Medicine and Pathology, University of Minnesota, Minneapolis, MN 55455, USA; nels2055@umn.edu; 4 Ruesch Center for the Cure of Gastrointestinal Cancers, Lombardi Comprehensive Cancer Center, Washington, DC 20007, USA; Benjamin.A.Weinberg@gunet.georgetown.edu; 5 Simmons Comprehensive Cancer Center, University of Texas Southwestern Medical Center, Dallas, TX 75390, USA; muhammad.beg@utsouthwestern.edu; 6 Department of Medical Oncology, Levine Cancer Institute, North Carolina, NC 28204, USA; Mohamed.Salem@atriumhealth.org; 7 Medicine, Norris Comprehensive Cancer Center, University of Southern California, Los Angeles, CA 90033, USA; lenz@med.usc.edu; 8 Department of Oncology, School of Medicine, Wayne State University, Detroit, MI 48202, USA; philipp@karmanos.org; 9 Division of Hematology/Oncology, Lifespan Cancer Institute, Department of Pathology & Laboratory Medicine, The Warren Alpert Medical School of Brown University, Providence, RI 02903, USA; wafik_el-deiry@brown.edu

**Keywords:** colon cancer, colorectal cancer, molecular oncology, oncogene, KRAS, A59T, immuno-oncology, predictive biomarkers

## Abstract

The molecular heterogeneity of *KRAS* is well established, with a pool of variants comprising >75% of all known mutations; this pool includes mutations in classic codons 12, 13, and 61, as well as 146 and 117. In addition, there are rare variants that are more frequently encountered clinically due to the advances in next-generation sequencing and more widespread implementation of All-*RAS* sequencing over the past five years. We have previously identified a missense variant of *KRAS*, A59T, in a patient with CRC that was associated with a response to an epidermal growth factor inhibitor when added to chemotherapy, supporting the hypothesis that distinct biochemical impacts of different *KRAS* mutations may produce varied responses to targeted therapy. In this study, we explored a large genomic database comprising 17,909 cases of CRC to determine the prevalence of the A59T mutation and characterized the concurrent genomic alterations associated with this variant in more detail, particularly in relation to the expanding set of potential predictive immuno-oncologic biomarkers. We identified 14 cases of A59 mutations in this dataset (0.08% prevalence). We evaluated the prevalence of high tumor mutation burden (TMB), positive PD-L1 expression, and microsatellite instability-high/mismatch repair-deficiency (MSI-H/dMMR) using both next generation sequencing (NGS) and immunohistochemistry (IHC). The genomic features of pertinent signaling pathways were also described, including *RAS* pathway, chromatin remodeling, DDR, hedgehog signaling, PI3K, receptor tyrosine kinases, signal transduction, TGF-beta, TP53, and WNT. We uncovered a high level of association of predictive markers of responsiveness to checkpoint inhibition and potentially other forms of immunotherapy, with nearly half of all cases harboring microsatellite instability as assessed using NGS. A59T was also detected in 11 additional cancer types, most prominently in cases of gynecologic or other gastrointestinal sites of origin. This study provides supportive evidence that A59T, and possibly other similarly rare *KRAS* variants, co-occur with predictive biomarkers of response to immunotherapy.

## 1. Introduction

The implementation of next generation sequencing that routinely assesses the full coding sequence of K-, N-, and H-*RAS* genes has demonstrated a larger, more diverse spectrum of cancer-associated *RAS* mutations than initially characterized in colon and other solid cancers with more focused molecular approaches. *KRAS* is mutated in 35–45% of colorectal cancers (CRC), and an additional 5–10% of cases harbor mutations in the *NRAS* oncogene; alterations in *HRAS* in CRC are very rare. Nearly all *KRAS* mutations are located in codons 12 and 13 [1]. The implications of improved identification of mutations in predominantly *KRAS,* as well as in *NRAS*, are significant for tailoring treatment options, as it is generally accepted that any *RAS* mutation creates a state of natural resistance to treatment with epidermal growth factor receptor (EGFR) inhibitors and is also associated with resistance to *BRAF* inhibitors in *BRAF*-mutated tumors.

In 2017 we reported a case of a patient with metastatic CRC that initially tested negative for *KRAS* codon 12/13 mutations, and the patient’s tumors had an objective and sustained clinical and radiologic response following the addition of the EGFR inhibitor panitumumab to the same chemotherapy combination [2]. The patient’s tumors were retested later on an NGS platform [3] and were found to harbor an uncommon missense mutation in *KRAS* (A59T; *KRAS:* c.175G>A (p.Ala59Thr)) [2,3]. The observation of significant clinical response despite the presence of a *KRAS* mutation with oncogenic biochemical features [2] was counterintuitive and led us to postulate that the mechanistic impact of this missense variant may in fact be distinct from other common *KRAS* mutations and permit tumor response to EGFR inhibition. This hypothesis was countered by an accompanying editorial to our report by Loree and Kopetz [4], who posited that the intratumoral heterogeneity of *KRAS*, in which the patient’s tumors harbored both wild-type *KRAS*-expressing cells and cells expressing the detected A59T mutation, may have led to the response. Thus, we hypothesized that A59T, and possibly even other *KRAS* variants, may in fact be more sensitive to EGFR inhibition and/or other emerging validated markers of response to immuno-oncology (IO) modalities than previously believed. We thus sought to further characterize this unusual variant by exploring a large dataset of CRC with available all-*RAS* sequencing as well as extensive genomic profiling, including MSI-H status and tumor mutational burden (TMB).

## 2. Methods

A total of 17,909 colorectal adenocarcinoma tumors were analyzed by Caris Life Sciences (Phoenix, AZ, USA) as part of routine comprehensive molecular profiling. Next-generation sequencing (NGS) was performed on genomic DNA isolated from formalin-fixed paraffin-embedded (FFPE) tumor samples using either the NextSeq platform or the Illumina MiSeq platform (Illumina, Inc., San Diego, CA, USA). For the NextSeq platform, a custom-designed SureSelect XT assay was used to enrich 592 whole-gene targets (Agilent Technologies, Santa Clara, CA, USA). For the Illumina MiSeq platform, specific regions of 47 genes of interest related to cancer genomics were amplified using a modified Illumina TruSeq Amplicon Cancer panel. All variants were detected with >99% confidence based on allele frequency and amplicon coverage, with an average sequencing depth of coverage of >500 and an analytic sensitivity of 5%.

Prior to molecular testing, tumor enrichment was achieved by harvesting targeted tissue using manual microdissection techniques. The genetic variants identified were interpreted by board-certified molecular geneticists and categorized as ‘pathogenic’, ‘presumed pathogenic’, ‘variant of unknown significance’, ‘presumed benign’, or ‘benign’, according to the American College of Medical Genetics and Genomics (ACMG) standards. When assessing mutation frequencies of individual genes, ‘pathogenic’ and ‘presumed pathogenic’ were counted as mutations while ‘benign’, ‘presumed benign’ variants, and ‘variants of unknown significance’ were excluded.

TMB was measured (592 genes and 1.4 megabases [MB] sequenced per tumor) by counting all nonsynonymous missense mutations found per tumor that had not been previously described as germline alterations. The threshold to define TMB-high was greater than or equal to 17 mutations/MB [5].

Immunohistochemistry (IHC) was performed on full formalin-fixed paraffin-embedded (FFPE) sections of glass slides. The slides were stained using automated staining techniques, per the manufacturer’s instructions, and were optimized and validated per Clinical Laboratory Improvement Amendments (CLIA)/CAO and ISO requirements. The staining was scored for intensity (0 = no staining; 1+ = weak staining; 2+ = moderate staining; 3+ = strong staining) and staining percentage (0–100%). Results were categorized as positive or negative by defined thresholds specific to each marker based on published clinical literature that associates biomarker status with patient responses to therapeutic agents. A board-certified pathologist evaluated all IHC results independently. The primary antibody used against PD-L1 was SP142 (Spring Biosciences, San Francisco, CA, USA). The staining was regarded as positive if its intensity on the membrane of the tumor cells was ≥2+ (on a semiquantitative scale of 0–3: 0 for no staining, 1+ for weak staining, 2+ for moderate staining, or 3+ for strong staining) and the percentage of positively stained cells was >5%.

A combination of multiple test platforms was used to determine the microsatellite instability (MSI) or mismatch repair (MMR) status of the tumors profiled, including fragment analysis (FA, Promega, Madison, WI, USA), IHC (MLH1, M1 antibody; MSH2, G2191129 antibody; MSH6, 44 anti-body; and PMS2, EPR3947 antibody [Ventana Medical Systems, Inc., Tucson, AZ, USA]), and NGS (for tumors tested with NextSeq platform [6], 7000 target microsatellite loci were examined and compared to the reference genome hg19 from the University of California). The tumor was determined MSI-high (MSI-H) by FA if two or more mononucleotide out of the five markers included in the assay were abnormal; the tumor was considered mismatch repair deficient (dMMR) by IHC if complete absence of protein expression of any of the four proteins was observed; the tumor was considered MSI-H by NGS by a threshold of 46 or more altered loci per tumor. MSI or MMR status of the tumor was determined in the order of IHC, FA, and NGS.

This study was conducted in accordance with guidelines of the Declaration of Helsinki, Belmont report, and U.S. Common rule. In keeping with 45 CFR 46.101(b) (4), this study was performed utilizing retrospective, deidentified clinical data. Therefore, this study is considered IRB exempt and no patient consent was necessary from the subjects.

## 3. Results

Of 17,909 cases of CRC reviewed, we identified 14 cases of A59T mutations (0.08%), a prevalence that is consistent with other sources [7], including the American Association for Cancer Research (AACR) GENIE database (https://www.mycancergenome.org/content/alteration/kras-a59t/, accessed 24 September 2020). Demographics are reviewed in Table 1 (Demographics and Cohort specifics). Samples from 10 of the 14 cases were from primary tumors; the other four samples were obtained from metastatic tumors (from lung, liver, lymph node, and abdomen, not otherwise specified (NOS), respectively). In terms of colonic location of the primary tumor, there was a relatively equal distribution of tumors in terms of sidedness, five left vs. five right, and the others were either transverse of unclear anatomic location. Two of the five cases designated as left sided were located in the rectum.

**Figure 1:**
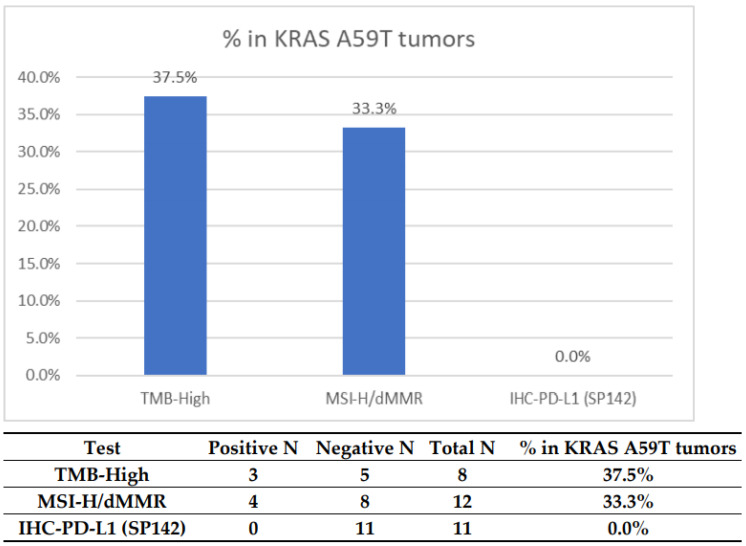
Prevalence of potential predictors for IO markers in *KRAS* A59T-mutated CRC. The cutoff for TMB high was >17. The cutoff for PD-L1 (SP142) was 2+, 5%.

We first investigated whether the presence of A59T mutations was associated with predictive markers of response to immunotherapy (IO). This analysis detected a notable enrichment of MSI-H/dMMR (37.5%) and TMB-H (33.3%); positive PD-L1 expression was not seen, consistent with current lack of clinical utility in testing this marker in CRC (Figure 1, Table 2). Among the 12 tumors with MSI/MMR status results available, four were MSI-H or MMR deficient; eight tumors were tested with more than one platform, and the results were concordant between the platforms.

**Figure 2:**
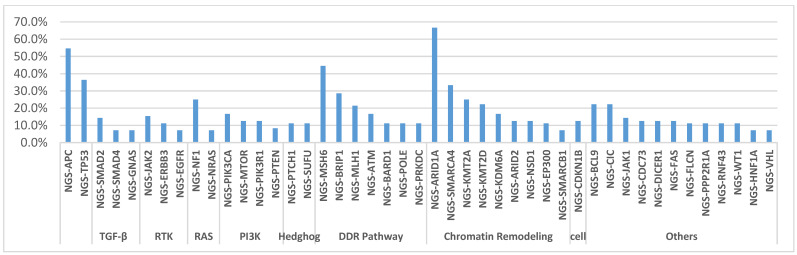
List of the most prevalent comutated genes in *KRAS* A59T mutant CRC tumors, categorized by pathway or role.

The A59T mutation was seen in concurrence with mutations in relevant molecular signaling pathways (Figure 2, Table 3 and Table 4). The most prevalent mutations were in those related to DNA damage repair, including MSH6 (44%), MLH1 (21.4%), and POLE (11%), or chromatin remodeling, including ARID1A (66.7%); all of these alterations were pathogenic or presumed pathogenic as determined by molecular geneticists, and no variants of unknown significance were taken into account. Interestingly, other comutant alterations included additional alterations in *NRAS* in 1 tumor, a rare A18T mutation (7.1%); PIK3CA in 2 tumors (16.7%); and EGFR in 1 tumor, also a rare D761N mutation (7.1%). Canonical mutations seen in CRC, including APC (54.5%) and TP53 (36.4%), were seen at high frequency. No clinically actionable gene fusions were identified by NGS in any of these cases.

We also examined our database for other, non-CRC malignancies harboring A59T mutations in *KRAS*. We detected 36 additional cases of A59T variants in tumors from patients with other cancers, including most prominently in endometrial, gastroesophageal, and ovarian carcinomas (Table 5). Interestingly, *KRAS* A59T variants are observed more frequently in MSI-H/dMMR tumors compared to their MSS/pMMR counterparts. These results demonstrate that A59T is not exclusive to colorectal cancers, but rather is also present in a spectrum of additional solid tumor malignancies in which *RAS* is implicated, and that the association with MSI-H/dMMR status exists in multiple tumor types. Overall, of these 36 non-CRC cases, 31/36 (86.1%) were derived from gynecologic or other gastrointestinal sites of origin.

## 4. Discussion

It has been established that oncogenic *KRAS* mutations stimulate a tumor-promoting inflammatory microenvironment that differentiates these tumors from their *RAS* wild-type counterparts [8]. In the context of our previous observation of an objective radiographic response in a patient with A59T-mutated CRC, we postulated that the genomic profile of these tumors includes markers associated with the response to IO-based treatments. Here, we examined an expansive database (>17,000 CRC tumors) to uncover 14 cases of the A59T missense mutation of *KRAS* and examined potential associations with clinically relevant biomarkers that predict responsiveness to immunotherapy. We did uncover a high level of association of predictive markers of responsiveness to checkpoint inhibition and potentially other forms of IO-based immunotherapy, with 4 of 12 cases harboring microsatellite instability (MSI-H), as assessed using a combination of various platforms. There was also a high extent of coassociation of A59T with alterations in ARID1A, a regulator of transcription implicated in chromatin remodeling that includes a DNA-binding domain which binds AT-rich sections of DNA; colorectal tumors with mutant ARID1A are characterized by higher rates of MSI-H and/or TMB-high status and T-cell infiltration [9].

The A59T missense mutation was confirmed to be very rare, consistent with other recent studies [10]; the prevalence in our study was only 14 out of ~17,000 cases. In general, little is known about the biochemistry and overall biology of this unique variant. What has been reported is that cells with A59T, as with a select few other forms of *KRAS* and *NRAS*, have higher rates of cell proliferation and migration in vitro; furthermore, A59T deregulates extracellular signal-related kinase (ERK) and the downstream target ETS transcription factor ELK1. Based on reports to date, it is clear that the role of A59T as a molecular regulator is quite different than other, more well-characterized and more common forms of *RAS* [11]. The A59T mutation reduces GTPase activity fivefold [12] and doubles the nucleotide exchange rate compared to wild-type *RAS*. It also eliminates interactions between *RAS* and the guanine nucleotide exchange factor SOS1 [13]. A59T is also noncanonical in *HRAS* [14] and has been shown to induce malignant transformation with high efficiency in mouse fibroblasts [15]; the nucleotide exchange rate of A59T-mutant *HRAS* has been reported to be up to ten times higher than the wild-type version of *HRAS*, concurrent with decreased GTPase activity [16,17]. In this study, we uncover for the first time the association of the *KRAS* A59T mutation with MSI-H/dMMR in CRC as well as in other cancer types, including endometrial cancer and gastroesophageal cancer. Overall, the unique structural biology and consequent downstream molecular effects of the A59T variant differ enough from other isoforms of *KRAS* to possibly explain potential increased susceptibility to EGFR inhibition. At the same time, the enrichment of IO markers in this subpopulation, combined with these altered effects secondary to the structural biology, provide a different landscape in terms of the clinical response to therapy.

Despite its rarity, the A59T mutation serves as a potential paradigm for variants of *RAS* that counter the notion that EGFR inhibition does not work at all against any cancer cells harboring any form of genetic *RAS* alteration. This variant was suggested as a negative predictor for EGFR-targeted agents in a randomized controlled trial which included only seven A59T mutant patients, in which retrospective analysis showed that when these seven patients were removed from the wild-type cohort, there was an improvement of the hazard ratio favoring the use of anti-EGFR therapy [18]. Prior meta-analysis and retrospective studies investigating exon 3 or 4, *KRAS* mutations included small numbers of A59T variants and suggested that, when treated with anti-EGFR therapies, exon 3- and 4-mutant *KRAS* variants behave similar to exon 2 variants. The mutation is now included in the guideline as part of the *RAS* mutations to determine the use of cetuxumab/panitumumab [19,20,21]. We previously reported that A59T tumors were responsive to EGFR inhibition in a single patient with metastatic chemorefractory colorectal cancer, challenging the one-size-fits-all approach for using *KRAS* mutation as a negative predictor for anti-EGFR therapy, and that observation stimulated this wider attempt at evaluation. Another recent study published a similar finding of the response of a *KRAS* A59T-harboring tumor to cetuximab, with the additional observation that this variant was enriched in tumors with low mutant allele frequencies [22]. For comparison, retrospective analysis of early studies evaluating the efficacy of EGFR inhibitors provided some evidence that patients with tumors harboring another *KRAS* isoform—G13D (glycine to aspartic acid at amino acid 13)—had a response to EGFR inhibition with cetuximab. However, subsequent prospective evaluation of patients with this molecular subset did not show evidence of significant increases in disease control to cetuximab with irinotecan, nor responses to single agent cetuximab [23]. Objective responses seen in some cases have been attributed at the molecular level to impaired binding of the G13D variant to Neurofibromin (NF1). The depletion of endogenous *RAS* activity through this altered binding may provide a crucial biochemical difference that leads some such tumors to behave biologically more like *RAS* wild type than like other RAS-mutant colorectal tumors [23,24]. A lesson learned from the investigation of G13D reinforces the concepts of tumor heterogeneity, the importance of understanding the biochemistry of *RAS* interactions, and the fact that the response of either G13D or A59T mutation cases is not likely to be ‘all or none’. What remains to be elucidated is what specific *RAS* pathway mutations are most affected by these uncommon variants and, in turn, how the natural biology, rate of cell proliferation, and other factors differ from that of other *RAS* isoforms.

When *KRAS* and MSI-H co-exist, the most common locations of these *KRAS* mutations are within codons 12 and 13 [25]. *KRAS* mutations and MSI-H are especially prevalent, together, in young-onset/early-onset forms of CRC. *KRAS* mutations are present in ~40% of CRC overall, but this number is significantly higher (~55%) in patients with young adult/early-onset forms of CRC [26]. We postulate that this disparity has critical implications for the notion that early-onset CRC has a different biologic imprint and thus clinical behavior than CRC that occurs in older adults. This is especially important in young-onset CRC because of the role of mutated *RAS* in the early stages of CRC carcinogenesis in young adults (30–50), proposed by Vogelstein 3 decades ago [27]. New therapies that target the biological mechanisms of metastatic CRC are urgently needed to improve outcomes among patients whose disease is chemoresistant. The A59T mutation may be enriched in this young adult/early-onset CRC population, although the numbers are relatively too few to assume this too firmly; it is also difficult to make this connection in our dataset, as the median age was older (>60). If this mutation were to be found to be enriched in this subpopulation, then that finding would underline additional genomic differences that may affect the efficacy of anti-EGFR or other therapeutic agents. Additional advances and more universal implementation in All-*RAS* testing may uncover more cases than identified to date.

The presence of MSI-H and/or TMB has gained importance in the past several years, independent of *RAS* status, largely due to improvements in testing methods, including next-generation sequencing, but even more so due to their therapeutic implications. Microsatellite instability is known to lead to an accumulation of mutations, including frameshifts as well as point mutations in CRC driver genes; however, the association with *KRAS* point mutation A59T has not been previously reported [28]. The identification of relatively high TMB values has been associated with the potential therapeutic response to checkpoint inhibitors in some solid tumor types [29], leading to approval by the U.S. Food and Drug Administration in June 2020 of pembrolizumab for both adult and pediatric patients with chemorefractory solid tumors harboring TMB ≥ 10 mutations/megabase (mut/Mb) [30]. Although use of TMB as a marker for this purpose remains somewhat controversial [31,32,33], there may be subgroups of TMB-high tumors that in combination with other driving mutations may provide better predictive value than TMB status alone. Thus, despite the relative rarity of the A59T mutation, the enrichment of TMB and MSI-H/dMMR may open the door to strategies for identifying more specific subpopulations that could benefit the most from pembrolizumab and other IO therapies to come. NGS has been shown to be an optimal approach broadly for assessing MSI in solid tumor histologies. Hause et al. have elucidated that MSI signatures reside within the context of differences dependent on surrounding cancer-associated driver mutations and also responses to selective pressures, including the potential ability of MSI to induce cancer-driving mutations [34]. Considering the finding of enrichment, or at least association, of A59T with MSI-H cases detected in our analysis, A59T may be one of those variants, and that finding may serve as a paradigm for this evolution during the earliest and middle stages of carcinogenesis of such tumors.

One of the downstream effects of oncogenic *RAS* in general is increased PD-L1 expression, which in turn promotes an immunoresistant phenotype [35]. However, in our study, PD-L1 was not a factor as it was not associated with the A59T cases we identified. This finding may not be surprising in light of previous data demonstrating inverse associations of PD-L1 expression with *KRAS* mutations in CRC, particularly in MSI-H tumors [36]. The expression of PD-L1 across a spectrum of non-small-cell lung cancers (NSCLC) of varying *KRAS* mutant isoforms is predictive of responses to immune checkpoint inhibitors in a similar fashion to wild-type NSCLC [37]. Our sample size of A59T-mutant CRC cases was too small to investigate this possibility further.

In sum, A59T is an example of a relatively rare form of *RAS* mutation that merits investigation to challenge the paradigm that *RAS*-driven CRCs are unresponsive to treatment using EGFR inhibition or other similar therapeutic strategies. However, we observe overall a lack of concurrent driver events in the 14 tumors identified, suggesting a potential oncogenic role for the variant in CRC. The association with known markers potentially predictive of responses to immunotherapeutic intervention (e.g., dMMR/MSI-H) and chromatin remodeling (e.g., mutant ARID1A) underscores the notion that *RAS* is heterogeneous, and its activity is not uniform. It is more likely that tumors with the A59T variant and no evidence of dMMR/MSI-H or other coalterations, such as ARID1A, POLE, etc., behave biologically and clinically as one would expect from the general pool of mutant *KRAS* CRC tumors (i.e., resistant to EGFR inhibition). In terms of how these findings may have clinical relevance for oncologists who identify the A59T mutation in their patients with CRC, the evidence that anti-EGFR blockade may be effective in these patients remains limited to several reports of individual cases. Although the rarity of this mutation and thus the small sample size preclude definitive testing, special attention should be paid to ensure their tumors are assessed for MSI-H that will make them eligible for IO therapy following the progression on standard-of-care cytotoxic chemotherapies (FOLFOX and FOLFIRI). A high level of association with markers of an inflammatory microenvironment, such as mutant ARID1A, opens the door to investigation of the potentially distinct tumor microenvironment of these tumors and differences in their biologic and biochemical activity compared with tumors bearing other, more canonical isoforms of *KRAS*. In the burgeoning area of the investigation of the utility of immune checkpoint blockade in select cases of CRC, careful examination is needed to better identify such cases of A59T in combination with MSI-H, as well as to identify such candidates earlier for immunotherapeutic approaches, including on clinical trials.

## Figures and Tables

**Table 1:** Demographics and cohort specifics listed by sex and tumor location used for genomic assessment (primary vs. metastatic tumor) or sidedness of the primary tumor (*p* = 0.59 for median age of males vs. females).

	Total N (%)	Female N (%)	Male N (%)	Median Age Female	Median Age Male	Age Range
Primary	10 (71.4)	5 (35.7)	5 (35.7)	63.0	62.0	32–83
Metastatic	3 (21.4)	2 (14.3)	1 (7.1)	63.0	83.0	61–83
Unclear	1 (7.1)	0 (0.0)	1 (7.1)	NA	31.0	31
Left	5 (35.7)	3 (21.4)	2 (14.3)	50.0	48.5	31–68
Right	5 (35.7)	1 (7.1)	4 (28.6)	63.0	72.0	49–83
Transverse	1 (7.1)	1 (7.1)	0 (0.0)	63.0	NA	63
Unclear	3 (21.4)	2 (14.3)	1 (7.1)	63.0	62.0	61–65
**Total**	**14**	**7 (50)**	**7 (50)**	**63.0**	**62.0**	**31** **–83**

**Table 2:** Determination of MSI-H/dMMR status in the 14 *KRAS* A59T-mutated CRC tumors. MSI-H-dMMR status is determined by a combination of NGS, fragment analysis (FA), and IHC.

Case ID	MSI/MMR	NGS-MSI	FA-MSI	MMR Deficiency (Four IHCs)
1	Not Tested	Not Tested	Not Tested	Not Tested
2	Not Tested	Not Tested	Not Tested	Not Tested
3	MSS/pMMR	Not Tested	stable	proficient
4	MSS/pMMR	Not Tested	Not Tested	proficient
5	MSS/pMMR	stable	Not Tested	proficient
6	MSS/pMMR	Not Tested	stable	proficient
7	MSS/pMMR	stable	stable	proficient
8	MSS/pMMR	stable	stable	proficient
9	MSI-H/dMMR	MSI-H	Not Tested	deficient
10	MSI-H/dMMR	MSI-H	Not Tested	Not Tested
11	MSS/pMMR	stable	Not Tested	proficient
12	MSI-H/dMMR	MSI-H	Not Tested	deficient
13	MSS/pMMR	stable	Not Tested	proficient
14	MSI-H/dMMR	MSI-H	Not Tested	Not Tested

**Table 3:** List of identified prevalent mutations co-occurring with *KRAS* A59T mutations in CRC, sorted in the context of molecular pathways.

Pathway	Test (NGS)	Positive	Negative	Total	% KRAS MT A59T
WNT Signaling Pathway	APC	6	5	11	54.5%
TP53 Pathway	TP53	4	7	11	36.4%
TGF-β signaling pathway	SMAD2	1	6	7	14.3%
	SMAD4	1	13	14	7.1%
Signal Transduction	GNAS	1	13	14	7.1%
Receptor Tyrosine Kinases/Co-factors	JAK2	2	11	13	15.4%
	ERBB3	1	8	9	11.1%
	EGFR	1	13	14	7.1%
RAS Pathway	NF1	1	3	4	25.0%
	NRAS	1	13	14	7.1%
PI3K Pathway	PIK3CA	2	10	12	16.7%
	MTOR	1	7	8	12.5%
	PIK3R1	1	7	8	12.5%
	PTEN	1	11	12	8.3%
Hedgehog Signaling Pathway	PTCH1	1	8	9	11.1%
	SUFU	1	8	9	11.1%
DDR Pathway	MSH6	4	5	9	44.4%
	BRIP1	2	5	7	28.6%
	MLH1	3	11	14	21.4%
	ATM	2	10	12	16.7%
	BARD1	1	8	9	11.1%
	POLE	1	8	9	11.1%
	PRKDC	1	8	9	11.1%
Chromatin Remodeling	ARID1A	4	2	6	66.7%
	SMARCA4	3	6	9	33.3%
	KMT2A	2	6	8	25.0%
	KMT2D	2	7	9	22.2%
	KDM6A	1	5	6	16.7%
	ARID2	1	7	8	12.5%
	NSD1	1	7	8	12.5%
	EP300	1	8	9	11.1%
	SMARCB1	1	13	14	7.1%
Cell Cycle	CDKN1B	1	7	8	12.5%
Other biomarkers	BCL9	2	7	9	22.2%
	CIC	2	7	9	22.2%
	JAK1	1	6	7	14.3%
	CDC73	1	7	8	12.5%
	DICER1	1	7	8	12.5%
	FAS	1	7	8	12.5%
	FLCN	1	8	9	11.1%
	PPP2R1A	1	8	9	11.1%
	RNF43	1	8	9	11.1%
	WT1	1	8	9	11.1%
	HNF1A	1	13	14	7.1%
	VHL	1	13	14	7.1%

**Table 4:** List of mutations sorted for each of the 14 *KRAS* A59T CRC cases in our cohort.

Pathway	Tumor	1	2	3	4	5	6	7	8	9	10	11	12	13	14
	MSI Status	High	High	High	High	Low	Low	Low	Low	Low	Low	**Low**	**Low**		
	NGS-POLE	0	0	0	0	1	0	0	0	0					
	TMB per Mb	49	IND	14	19	294	5	16	11	9					
RAS Pathway	NGS-KRAS	A59T	A59T	A59T	A59T	A59T	A59T	A59T	A59T	A59T	A59T	**A59T**	**A59T**	**A59T**	**A59T**
	NGS-BRAF	0	0	0	0	0	0	0	0	0	0	0	0	0	0
	NGS-NRAS	0	0	0	0	A18T	0	0	0	0	0	0	0	0	0
Cell Cycle	NGS-CDKN1B	0	0	0		1	0	0	0	0					
Chromatin Remodeling	NGS-ARID1A	1	1	1	0				1	0					
	NGS-ARID2	0	1	0		0	0	0	0	0					
	NGS-EP300	0	0	0	0	1	0	0	0	0					
	NGS-KDM6A	1	0	0			0	0		0					
	NGS-KMT2A	0	1	0		1	0	0	0	0					
	NGS-KMT2D	1	1	0	0	0	0	0	0	0					
	NGS-NSD1	1	0	0		0	0	0	0	0					
	NGS-SMARCA4	1	1	1	0	0	0	0	0	0					
	NGS-SMARCB1	0	1	0	0	0	0	0	0	0	0	0	0	0	0
DDR Pathway	NGS-ATM	1	0	0		1	0	0	0	0	0	0	0	0	
	NGS-BARD1	0	0	0	0	1	0	0	0	0					
	NGS-BRIP1	0	1	1		0	0	0		0					
	NGS-MLH1	1	0	1	1	0	0	0	0	0	0	0	0	0	0
	NGS-MSH6	1	0	1	1	1	0	0	0	0					
	NGS-POLE	0	0	0	0	1	0	0	0	0					
	NGS-PRKDC	0	0	0	0	1	0	0	0	0					
Hedgehog Signaling Pathway	NGS-PTCH1	0	1	0	0	0	0	0	0	0					
	NGS-SUFU	0	0	0	0	1	0	0	0	0					
PI3K Pathway	NGS-MTOR	0	0	0		1	0	0	0	0					
	NGS-PIK3CA	0	0	0	1	1	0	0	0	0	0		0	0	
	NGS-PIK3R1	0	0	0		1	0	0	0	0					
	NGS-PTEN	0	0	0		1	0	0	0	0		0	0	0	0
	NGS-NF1	0				1	0			0					
	NGS-NRAS	0	0	0	0	1	0	0	0	0	0	0	0	0	0
Receptor Tyrosine Kinases/Co-factors	NGS-EGFR	1	0	0	0	0	0	0	0	0	0	0	0	0	0
	NGS-ERBB2	0	0	0	0	0	0	0	0	0	0	0	0	0	0
	NGS-JAK2	0	1	0		1	0	0	0	0	0	0	0	0	0
Signal Transduction	NGS-GNAS	0	1	0	0	0	0	0	0	0	0	0	0	0	0
TGF-β signaling pathway	NGS-SMAD2	0	0	0		1	0	0		0					
	NGS-SMAD4	0	0	0	0	1	0	0	0	0	0	0	0	0	0
TP53 Pathway	NGS-TP53	1	0	0	0	0	1	1	1	0		0		0	
WNT Signaling Pathway	NGS-APC	0	0	1	1	1	1	1	0	1			0		0
Other biomarkers	NGS-BCL9	1	1	0	0	0	0	0	0	0					
	NGS-CDC73	0	0	0		1	0	0	0	0					
	NGS-CIC	0	1	1	0	0	0	0	0	0					
	NGS-DICER1	0	0	0		1	0	0	0	0					
	NGS-FAS	0	0	1		0	0	0	0	0					
	NGS-FLCN	0	1	0	0	0	0	0	0	0					
	NGS-HNF1A	0	0	0	0	0	0	0	0	1	0	0	0	0	0
	NGS-JAK1	1	0	0		0	0	0		0					
	NGS-PPP2R1A	1	0	0	0	0	0	0	0	0					
	NGS-RNF43	0	1	0	0	0	0	0	0	0					
	NGS-VHL	0	0	1	0	0	0	0	0	0	0	0	0	0	0
	NGS-WT1	0	0	0	1	0	0	0	0	0					
	CNA-FGFR4	0	0		0	0	0	0	1	0					

1 = pathogenic variant/likely pathogenic variant; 0 = WT/benign/likely benign/VUS, or unclassified (blank boxes)

**Table 5:** List of additional cancer types and cases identified as harboring the A59T variant of *KRAS*.

Cancer Types	N	MSI-H/dMMR	MSS/pMMR	Unknown
Endometrial	18	10	5	3
Esophageal Junction	5	3	2	
Ovarian Surface Epithelial Carcinomas	4	2	1	1
Cancer of Unknown Primary	2	2		
Bladder	1			1
Cervical Cancer	1	1		
Cholangiocarcinoma	1	1		
Gastric Adenocarcinoma	1	1		
Lung Non-small cell lung cancer (NSCLC)	1		1	
Penile Cancer	1			1
Small Intestinal Malignancies	1	1		
**Total**	**36**	**21**	**9**	**6**

## Data Availability

The data presented in this study are available on request from the corresponding author. The data are not publicly available due to data size and privacy.
